# Building a Precision Medicine Delivery Platform for Clinics: The University of California, San Francisco, BRIDGE Experience

**DOI:** 10.2196/34560

**Published:** 2022-02-15

**Authors:** Riley Bove, Erica Schleimer, Paul Sukhanov, Michael Gilson, Sindy M Law, Andrew Barnecut, Bruce L Miller, Stephen L Hauser, Stephan J Sanders, Katherine P Rankin

**Affiliations:** 1 UCSF Weill Institute for Neurosciences University of California, San Francisco San Francisco, CA United States

**Keywords:** precision medicine, clinical implementation, in silico trials, clinical dashboard, precision, implementation, dashboard, design, experience, analytic, tool, analysis, decision-making, real time, platform, human-centered design

## Abstract

Despite an ever-expanding number of analytics with the potential to impact clinical care, the field currently lacks point-of-care technological tools that allow clinicians to efficiently select disease-relevant data about their patients, algorithmically derive clinical indices (eg, risk scores), and view these data in straightforward graphical formats to inform real-time clinical decisions. Thus far, solutions to this problem have relied on either bottom-up approaches that are limited to a single clinic or generic top-down approaches that do not address clinical users’ specific setting-relevant or disease-relevant needs. As a road map for developing similar platforms, we describe our experience with building a custom but institution-wide platform that enables economies of time, cost, and expertise. The BRIDGE platform was designed to be modular and scalable and was customized to data types relevant to given clinical contexts within a major university medical center. The development process occurred by using a series of human-centered design phases with extensive, consistent stakeholder input. This institution-wide approach yielded a unified, carefully regulated, cross-specialty clinical research platform that can be launched during a patient’s electronic health record encounter. The platform pulls clinical data from the electronic health record (Epic; Epic Systems) as well as other clinical and research sources in real time; analyzes the combined data to derive clinical indices; and displays them in simple, clinician-designed visual formats specific to each disorder and clinic. By integrating an application into the clinical workflow and allowing clinicians to access data sources that would otherwise be cumbersome to assemble, view, and manipulate, institution-wide platforms represent an alternative approach to achieving the vision of true personalized medicine.

## Introduction

Precision medicine holds the potential to revolutionize medicine [1-3], just as prior technological advances, such as microscopy, molecular diagnostics, and imaging, have done in the past. In the research realm, big data and artificial intelligence have yielded substantial advances that showcase the potential of precision medicine [4,5]. However, translating these advances into the clinical realm remains a challenge [6,7]. A patient is more likely to interact with complex algorithms informed by big data in the waiting room (ie, algorithms in the form of internet searches, travel directions, or tailored social media) than in the actual clinic. The medical field needs similarly intuitive interfaces that can collate the necessary patient-related data to highlight salient knowledge, pinpoint a patient’s condition, predict optimal therapy, or estimate the risk of disease or death [3]. Much of the required physical infrastructure is already in place, with computers being available in most clinics and the majority of clinical data being stored in electronic health records (EHRs). A small minority of wealthier clinics and health care systems have built custom, domain-specific interfaces into their EHRs to deliver the more complex precision medicine algorithms and visualizations that their physicians need; however, in the majority of health systems, only the most basic algorithms (eg, those for calculating BMI) are built into the EHR, while other, more sophisticated clinical indices (eg, atrial fibrillation stroke risk [8,9]) are calculated via manual entry into a public website [10].

**Figure 1:**
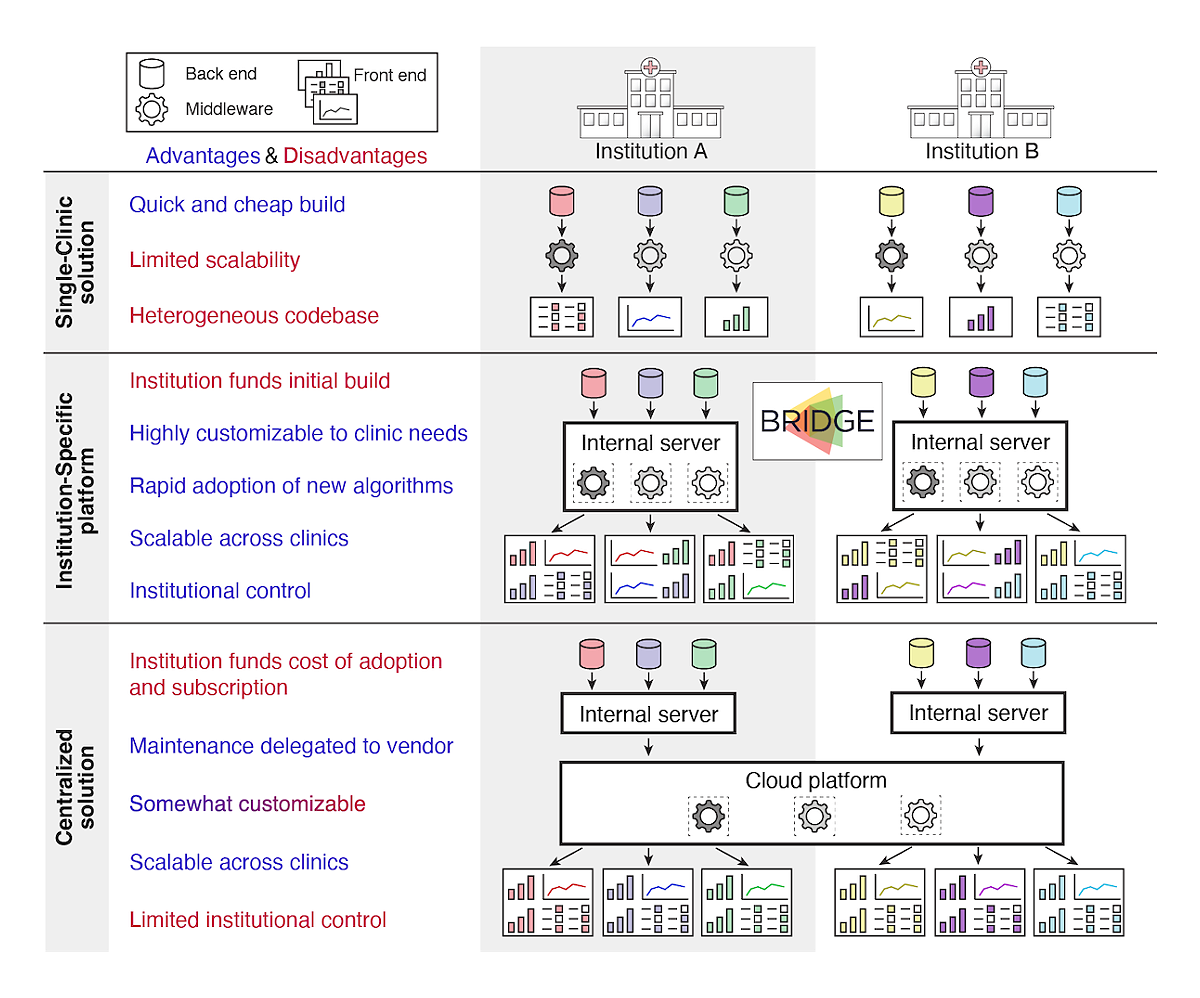
Approaches to delivering precision medicine results to the clinic. This figure compares the platform design elements across the following three main approaches to building clinical systems to support precision medicine: (1) single-clinic solutions, (2) institution-specific platforms like BRIDGE, and (3) centralized solutions purchased from external vendors. Key advantages (blue) and disadvantages (red) of each approach are listed.

The task of translating innovative precision medicine tools from research projects to clinical care is inhibited by a catch-22 problem. To justify the expense of building the costly computational infrastructure required to run complex algorithms on patient data, the algorithms or visualizations need to demonstrate real-world value. However, to evaluate and prove these algorithms’ value, the needed infrastructure must already be in place. One solution to this conundrum is building boutique, single-clinic solutions consisting of carefully designed, specialized algorithms or data displays built within or alongside the EHR [11,12]. Although this bottom-up approach is limited in scope to a single clinical domain and thus can be comparatively quick and cost-effective to implement, scalability and rapid obsolescence are major concerns. To adapt data displays to other clinics, an institution has to maintain, secure, and update an ever-expanding heterogeneous code base across those clinics. Yet, the originating “owners” of these algorithms are often clinical researchers and physicians without the backing of an enterprise-level developer team that is equipped to manage the software as a service over several years of use (Figure 1). The opposite extreme is commercial vendors building generalized health care software suites that run on cloud-based infrastructures. Such centralized solutions address the scalability challenges of bottom-up approaches, but the emerging health system–wide products are typically far too generic to meet the medically heterogeneous and shifting requirements of individual clinics. Furthermore, adopting such solutions requires substantial institutional investment, and becoming locked into a single vendor in a rapidly evolving marketplace poses a risk.

**Figure 2:**
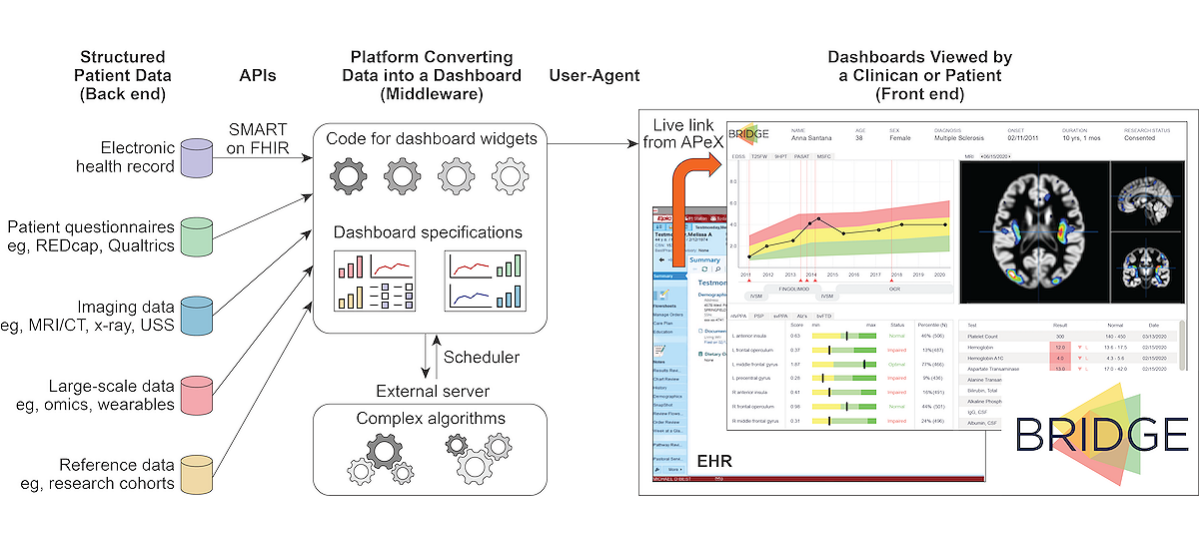
Overview of technological components for the integrated delivery of precision medicine through an institution-specific platform like BRIDGE. The flow of information is depicted as it moves from back-end data sources, through the integrating middleware layer of light and heavy computational resources, and to the multiple, functionally distinct widgets shown in a user-facing front-end dashboard designed to reflect the needs of a single clinic. APeX: Advanced Patient-Centered Excellence; API: application programming interface; CT: computerized tomography; EHR: electronic health record; FHIR: Fast Healthcare Interoperability Resources; MRI: magnetic resonance imaging; REDCap: Research Electronic Data Capture; SMART: Substitutable Medical Applications and Reusable Technologies; USS: ultrasound sonography.

Between these two extremes exists a third solution that solves many of the aforementioned problems. Institution-wide platforms permit rapid innovation in parallel across multiple clinics but are built on a single secure, stable, and cost-efficient technological foundation. These platforms benefit from a common architecture built within an institutional firewall with real-time EHR access and application programming interfaces (APIs) to major (eg, REDCap [Research Electronic Data Capture; Vanderbilt University] and Radiology PACS [Picture Archiving and Communication System]) and custom data resources, which facilitate the integration of multimodal research data across all specialties. Yet, these platforms also incorporate clinic-specific visualization tools that allow clinicians to tailor the display of information. Therefore, specific research discoveries can be rapidly translated into clinical tools that fit each specialty (Figure 2). This approach strikes a balance between the fast development and flexibility of single-clinic solutions and the scalability and sustainability of centralized health care solutions while optimizing transparent institutional oversight.

The BRIDGE platform at University of California, San Francisco (UCSF), is one example of this approach. Based on our experience with developing BRIDGE, we describe key considerations and practical steps for implementing institution-wide solutions in this rapidly progressing field to provide a road map for other health care systems considering a similar approach. We also consider future developments that will enable the medical community to quickly and comprehensively realize the potential of computational medicine to improve the lives of patients.

## Consideration 1: Human-Centered Design

### Overview of the Human-Centered Design of Precision Medicine Tools

For a precision medicine tool to be adopted in a clinic, it needs to provide pertinent, actionable information in a format that is appropriate to the user (either a clinician or a patient). Therefore, perhaps the most essential components of effective precision medicine tool deployment are the principles and phases of human-centered design (HCD) [13-15]. For tools targeted at medical professionals, clinician users, who are well informed, should be at the center of decisions about which technological format is the most appropriate for their workflow, which innovations in their specialty are scientifically ready for deployment at clinics, and how evaluations of tool effectiveness should be conducted to justify the continued use of such tools (Textbox 1). Many of these decisions reflect the dimensions of precision medicine, as articulated in a recent scoping review [7].

**Key decisions in designing a digital application for clinical research.**

**Key questions**

1. Who are the users (eg, clinicians, patients, and specialists)?
2. What do the users need (eg, novel data sources, novel algorithms, novel visualization, and data collation)?
3. How will it improve care (eg, patient experience, clinic efficiency, morbidity, and mortality)?
4. How does the user access the application (eg, individual log-in and authorization via an existing clinical system)?
5. Where is it hosted (eg, local server, cloud-based server, or external vendor)?
6. What is the maintenance schedule (eg, 9 AM to 5 PM on Monday to Friday or 24 hours per day year-round)?
7. What are the constraints of the system? For example, will it not write to the electronic health record? Are data behind an institutional firewall?

### Practical Considerations From the BRIDGE Experience

From its inception, BRIDGE exemplified both the principles and phases of HCD [13,14]. It was conceptualized and designed according to the requirements of clinician scientists, including the project’s principal investigators (manuscript authors RB, KPR, and SJS). Further, the key architectural decisions (Textbox 1) were made by applying HCD principles to engage clinician, patient, scientific, programming, design, industry, and institutional stakeholders.

**Figure 3:**
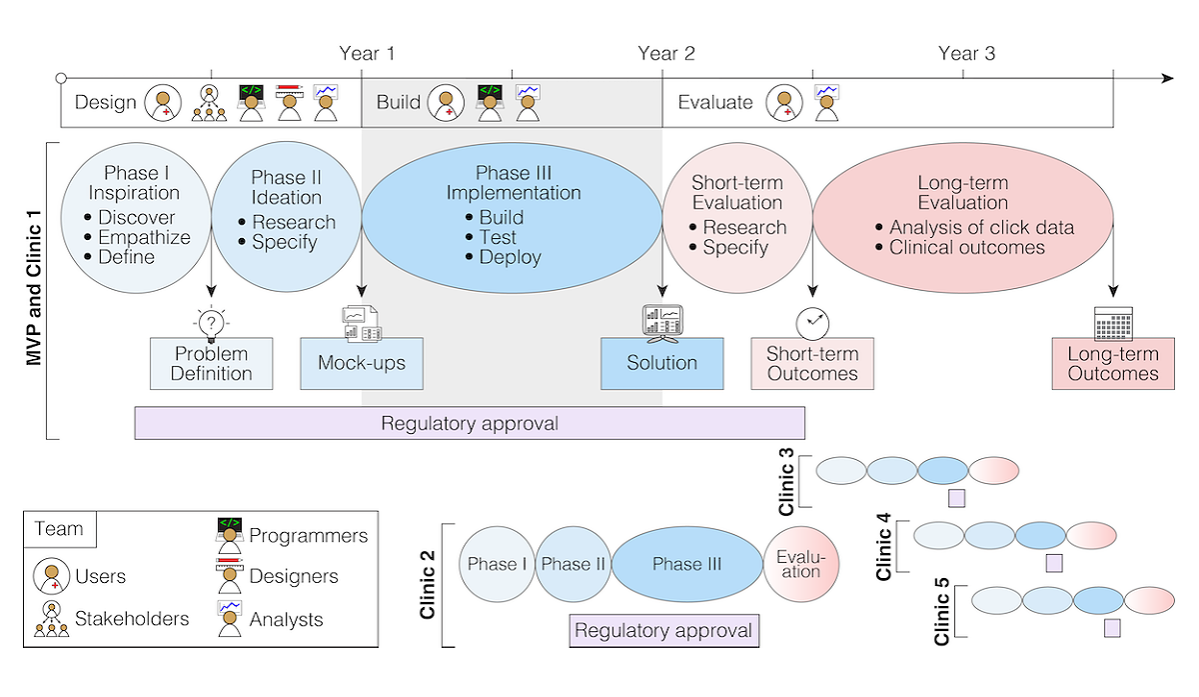
Example timelines and milestones of clinical application development. The design and development of an institution-specific platform for clinical applications, such as BRIDGE, is a multiyear undertaking (eg, 2 years for the MVP and clinic 1). Following the principles and phases of human-centered design ensures the development of a product that meets the needs of the users and the requirements of the institution. Obtaining institutional regulatory approval—a process that runs in parallel with the design and development processes—is critical and can risk becoming the rate-limiting step. The evaluation of the product initially focuses on user experience, followed by clinical outcomes such as morbidity, mortality, or efficiency. With the majority of the platform built, the design and build times are dramatically reduced for clinic 2, and they continue to fall as the process becomes refined (eg, under 6 months for clinics 3-5) and occurs in parallel. MVP: minimum viable product.

The three HCD phases are also being deployed in the iterative process of adapting BRIDGE to each new clinic that is interested in a BRIDGE dashboard (Figure 3). In the “Inspiration” phase, the BRIDGE clinician scientists and programmers identify and meet with a small number of clinician champions to collaboratively define the problems to be solved to improve care in the clinician champions’ clinic. They also generate ideal use cases based on that clinic’s workflow, such as those for data types, data sources, and visualizations. In the “Ideation” phase, a design mock-up is shared with a broader set of intended stakeholders from that clinic to obtain their input, after which the final set of minimum viable product (MVP) specifications is derived for the dashboard, and programming begins for the jointly approved design mock-up. The finalized MVP is built in the “Implementation” phase, during which early testing is conducted by a small superuser group of clinicians who generate feedback about bugs and minor refinements. These clinic domain experts are the primary drivers for designing and conducting formal evaluations of their precision medicine tools, which include clinician users’ feedback about dashboard ease, utility, and fidelity; patients’ satisfaction with care; impacts on workflow, including automated click tracking; and longer-term analyses of the clinical impact, value, and cost-effectiveness of these tools. Clinical validation, technological or therapeutic innovation, or user demand may result in further cycles of design.

### HCD: Future Directions

Since the back-end infrastructure of an institution-wide platform is unified, only 1 set of corresponding regulatory approvals is needed, and this approach reduces the cost and time required to develop a front-end tool and allows multiple tools to be developed in parallel (Figure 3). However, given the number of medical specialties, clinical scenarios, disorders, and algorithms across a health care system, engaging in this intensive HCD process with each new clinic will not be cost-effective in the long term. Instead, a library of existing data sources and graphical interfaces could be generated, and clinicians (or patients, ie, in the event of a patient-facing version) could customize this library to design their own dashboards, thereby freeing programmers to concentrate on developing new modular interfaces and data sources. Generating more universal standards for describing clinical dashboards and their connections to APIs and EHRs could ease the deployment of dashboards across a wide range of health care platforms. Containerization, the Substitutable Medical Applications and Reusable Technologies (SMART) on Fast Healthcare Interoperability Resources (FHIR) API, and the Epic App Orchard (Epic Systems) represent important steps in this direction, but substantial scope for further standardization remains. The adoption of this type of adaptable clinical dashboard at scale would provide sufficient data for iteratively testing and improving performance, resulting in a second data-driven evaluation phase that focuses on surveys and click data. As the scale of data grows, especially across institutions, a third design phase that is based on both clinical outcomes and user experience will become possible.

## Consideration 2: Technological Design

### Common Approaches to the Technological Design of Digital Health Tools

The architecture of most digital health tools involves a connection among the back-end databases, middleware software algorithms that convert the data into useful knowledge, and front-end displays for users (Figure 2). Both single-clinic and centralized solutions are often hard-coded to represent a specific data source and visualization type, which slows the development of novel iterations and results in higher overall costs. A more efficient solution is to build a framework of reusable APIs that connects a multiplying number of data sources, computational algorithms, and modular visualization schematics and is adaptable and scalable to diverse types of medical data and clinical specialties.

### Practical Considerations From the BRIDGE Experience

#### Overview of Practical Considerations

The BRIDGE platform was designed as a proof-of-principle MVP scaffold that could be developed efficiently and quickly but later refined and scaled up depending on its success and the collaborative opportunities generated. The HCD process made clear the following four key technical requirements: (1) it had to permit access to a variety of data sources (ie, beyond the EHR), which could then be either displayed directly or processed through computationally intense algorithms [7]; (2) it needed to enable the visualization of these data in an intuitive and actionable manner, and this process needed to be embedded in the clinical workflow, so that it was not cumbersome for clinicians to access or operate; (3) following logically from the second requirement, it required the ability to launch directly from the EHR; and (4) it had to be as modular as possible to make iterative clinic-by-clinic customizations easier and more efficient to program.

#### Data Sources

Many data types contribute to precision care. To build a data foundation for BRIDGE that would best meet the needs of a variety of clinical use cases, we opted to include real-time clinical data from the EHR, minimally processed data from widely available data platforms (REDCap and Qualtrics [Qualtrics International Inc]) [16], data from institutional tools (eg, TabCAT [Tablet-Based Cognitive Assessment Tool; UCSF]) [17] and research databases [18], and complex data that either cannot be currently hosted in the standard EHR or require processing by complex analytics processing pipelines (Figure 2). For example, images from the Radiology PACS can be obtained ahead of time based on scheduled appointments, thereby allowing time for computationally intensive image processing pipelines to run prior to a patient appointment. Further innovations requiring advanced data processing include accessing expansive knowledge networks to compute precise clinical risk and treatment predictions [19]. As it would represent the convergence of so many sensitive data streams, BRIDGE required robust front-end and back-end architectures that were unified around security and hosted within the UCSF firewall.

#### Workflow Fit and EHR Integration

As a fundamental requirement for BRIDGE, to give clinicians actionable information during patient encounters, it had to launch directly from patients’ records in the EHR (ie, Epic; Epic Systems) and pull their clinical data in real time (Figure 2). This resulted in a central technical decision to design BRIDGE as a SMART on FHIR application. Launching from the EHR resulted in additional clinical workflow benefits; discrete data could be collected at the point of care by using clinic-specific EHR Flowsheets and SmartForms (sharable across institutions), and data could then be pulled into clinical notes. Direct flowsheet data entry also allows BRIDGE to call and visualize discrete research data during clinic visits more efficiently. Enabling this launch functionality required interactions with the EHR development group and resources for funding their modifications to the EHR.

#### Modular Design

BRIDGE was designed to capitalize on a common language of clinical information flow through the creation of core widgets, or visualization modules, that can be adapted to an expanding array of clinical scenarios (Figure 2). At the time of BRIDGE MVP deployment, we had programmed the following four reusable core widgets: (1) longitudinal clinical course in the context of treatment, (2) cross-sectional metrics, (3) specialty-focused laboratory data, and (4) quantitative neuroimaging. Both the cross-sectional and longitudinal widgets allow patients’ scores and metrics to be contextualized against a larger reference cohort that indicates both normal and abnormal values as well as percentile calculations, thus allowing a patient’s clinical status to be interpreted by a clinician at a glance (Figure 2). We were able to convert existing precision medicine tools, such as the UCSF Multiple Sclerosis BioScreen longitudinal viewer [12] and the UCSF Brainsight magnetic resonance image processing and visualization tool [20] into these initial BRIDGE widgets. The configuration data for all viewers are stored by BRIDGE, which queries these data in real time and then renders the specified widgets and data sources for the clinician. Updates to the configuration can be made quickly when existing dashboards need to be adapted, thus enabling both ongoing user engagement and rapid deployment to meet the evolving needs of specific clinics. As we expand to other clinics, new widgets (eg, geolocation and genomics) that can be retroactively made available to existing clinics are being developed.

### Technological Design: Future Directions

Two architectural changes can be made. The first is integration with a middleware platform. BRIDGE is currently connected to multiple data sources through direct API integrations, and connecting to additional APIs necessitates the modification of the codebase. Making use of a platform that aggregates APIs would reduce maintenance efforts and promote more stable platforms. Examples of such platforms already exist (eg, Human API [21]) and include EHR data. The second architectural change is creating a graphical user interface (GUI) that clinicians can use to create their own dashboards. Currently, dashboard configuration is done by the BRIDGE development team. Building a GUI that allows clinicians to configure and customize their dashboards would accelerate progress and allow clinicians without programming experience to access relevant data sources. Such an endeavor will likely require the integration of institution-wide platforms and centralized platforms, and such an integration will benefit both types of platforms. The resulting unified platforms would probably combine generic, cloud-based back-end and middleware components but be able to deliver the customized, clinic-specific, front-end dashboards designed by clinicians through the GUI. Overall, BRIDGE aims to augment—not supplant—the EHR; should an institution’s visualization show clinical value, the institution could choose to maintain it in BRIDGE or integrate it into their EHR more permanently.

Innovations are also needed to improve data quality in the EHR, including tools that systematically flag likely data entry errors, simplify the correction of the EHR by a clinician, and ensure that corrections are distributed to all clinical tools. Finally, to demonstrate that these tools comply with the Health Insurance Portability and Accountability Act (HIPAA) or equivalent guidelines, a cross-institutional body that is responsible for testing and validating these solutions could be created. It might accelerate progress substantially by, for example, supporting cloud-based, HIPAA-compliant, off-the-shelf solutions to ease this data quality burden.

## Consideration 3: Regulation and Policy

Launching a clinical application with real-time access to identified patient health data requires close institutional oversight and multiple stages of regulatory approval, especially in cases where clear institutional road maps or leadership structures are lacking due to the innovative nature of such applications.

### Practical Considerations From the BRIDGE Experience

With regard to developing the BRIDGE MVP, the Epic EHR, and the SMART on FHIR application, technological capabilities were already available within our institution, but multiple security, privacy, technological, and compliance concerns had to be addressed. Specifically, authorizing an expandable, cross-specialty, modular platform rather than a domain- and clinic-specific tool was entirely novel. This necessitated parallel revisions to the approval process itself. Early in the design process, we set clear functional constraints that would reduce the barriers to institutional approval. Foremost among these were (1) conceptualizing BRIDGE as a clinical research tool that is custom designed with clinical specialists rather than as an institution-wide, enterprise-level clinical solution; (2) not requesting write access to the EHR (real-time read access was enabled); and (3) ensuring that data do not leave the institutional firewall. With an approved clinical research platform in place, the bar for institutional approval is substantially reduced for subsequent clinical dashboards that iterate on the initial design, reducing this multi-month process to a simple, clinic-specific sign-off (Figure 3). Further approval is required for applications that add novel functionalities or revisit one of the major systems constraints (eg, sending data to an external server).

### Regulation and Policy: Future Directions

BRIDGE provides a mechanism for rapidly deploying and evaluating novel precision medicine algorithms and visualizations developed by clinical researchers [22-24] to evaluate their clinical benefit [25]. As the system expands and more clinical visualizations become the standard of care, medical centers may eventually choose to move the fundamental infrastructure of their institution-wide platforms from an MVP clinical research entity, such as BRIDGE, to a full, enterprise-level clinical system that delivers the same capabilities at a higher level of reliability [26,27]. This shift will be precipitated by a number of considerations, including the need for professional-level version control and releases; automated testing and quality control; the capacity for multilevel monitoring, logging, and auditing; and the ability to handle high user volumes without concurrency issues. The institution will also need to ensure that there is adequate personnel infrastructure behind the system to permit sustainable 24-hour user support and timely design and adaptation for new clinics. In the end, all stakeholders must be able to trust the reliability and clinical value of the final platform and the sustainability of the system supporting it [28]. For many such algorithms, moving along the continuum from clinical research to enterprise clinical care may well necessitate regulatory approval from the Food and Drug Administration Center for Devices and Radiological Health [29], as spelled out in their Digital Health Innovation Action Plan, and alignment with the international Software as a Medical Device guidelines through the International Medical Device Regulators Forum.

## Consideration 4: Evaluation and Impact

### Pathway to Evaluation

Technological innovations in health care will ultimately be evaluated in terms of their impacts on patients, clinicians, data, and payors. In the near term, this requires the evaluation of a tool’s interpretability and fidelity, that is, whether clinicians and patients like, understand, and use the tool and whether the use of the tool improves patients’ experiences within the health system [15,28,30]. Making even the most complex algorithms visually digestible and actionable will be a key evaluation criterion [3]. To this end, for each BRIDGE dashboard, prior to measuring its clinical impact, we ensure that it meets key drivers of clinical adoption. We use the Health Information Technology Usability Evaluation Model [31] to evaluate at least 15 patients’ and 8 clinicians’ perceptions on the usefulness [32,33], ease of use [32,33], actionability [31], and likability [34] of each clinical dashboard. Low-scoring items (ie, <80% of respondents state “agree” for any given metric) engender another round of iterative development.

### Evaluation and Impact: Future Directions

**Figure 4:**
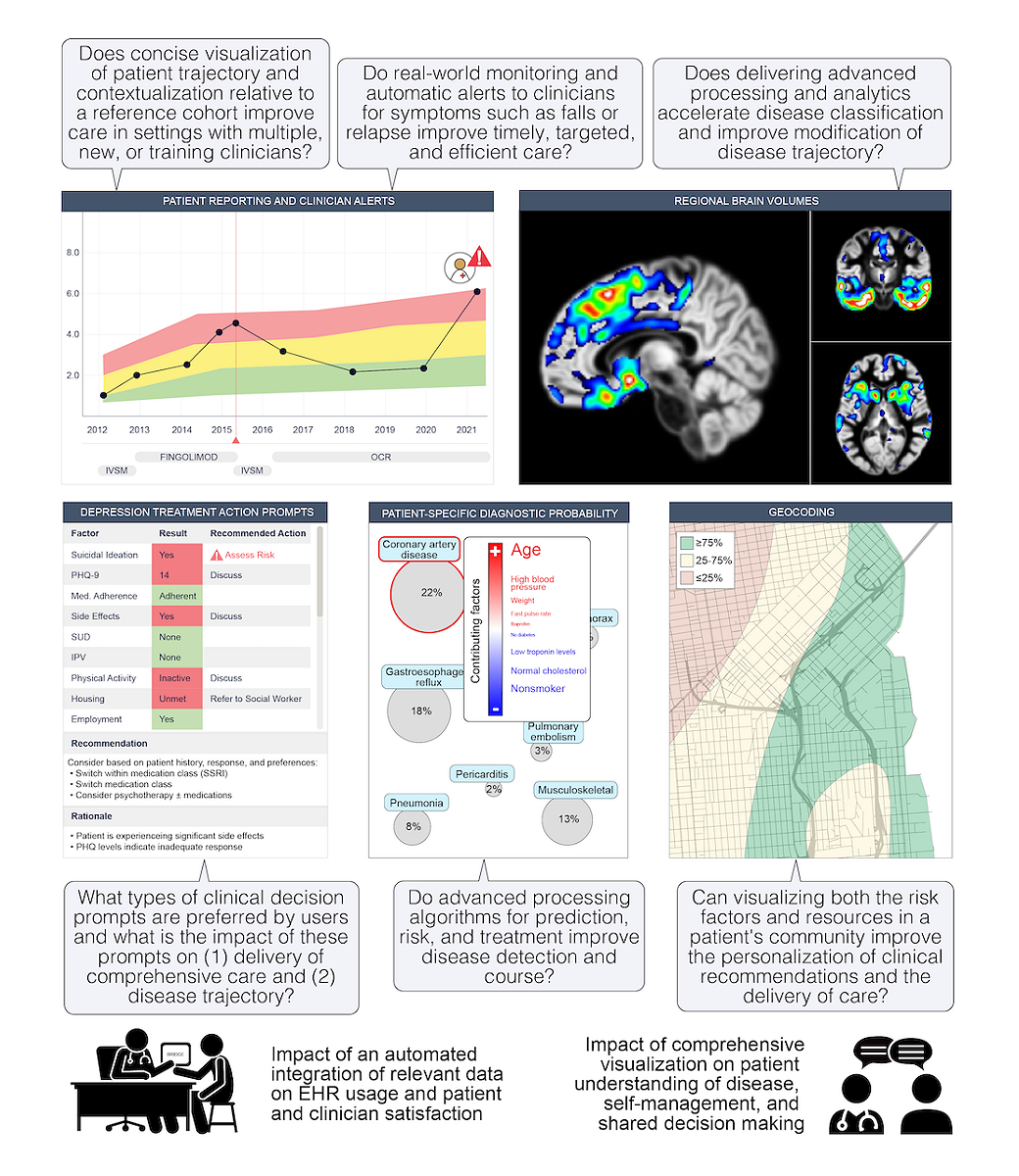
Prototypes of clinical research enabled by BRIDGE. Clinical research applications include impacts on patient-doctor interactions and clinical workflows; the impact of monitoring patient-generated data, such as patient-reported outcomes and activity monitoring data; and the impact of delivering more advanced image processing and clinical algorithms (including prediction, prevention, and treatment algorithms) into the hands of clinicians. EHR: electronic health record; IPV: intimate partner violence; IVSM: intravenous solumedrol; MVP: minimum viable product; OCR: ocrelizumab; PHQ: Patient Health Questionnaire; PHQ-9: Patient Health Questionnaire-9; SSRI: selective serotonin reuptake inhibitor; SUD: substance use disorder.

The impact of a dashboard like BRIDGE on clinical research and, eventually, care can be evaluated through in silico trials for answering a variety of clinical questions, as depicted in Figure 4. A near-term goal may be to compare users’ preference for 2 types of symptom displays or to evaluate the impact of BRIDGE on workflow efficiency (eg, determining whether the use of the tool reduces the overall time spent on “clicking” through a patient’s chart). Medium-term goals may be to refine a series of treatment action prompts that could yield a clinical decision support tool or to compare the effects of 2 different prediction algorithms on the risk of rehospitalization after a cardiac event. Long-term, altering clinical outcomes [2,25,27] that have obvious implications for health economics, such as reductions in the time to accurate diagnosis, rehospitalization, disability progression, morbidity, or death, will be directly relevant to an institution’s assessment of a tool’s utility.

## Discussion

Determining whether big data analytics will truly disrupt clinical care depends on providing clinicians with access to the results of these analytics. In this paper, we describe one approach to overcoming the technical hurdle of making algorithms clinically available: the development of BRIDGE, an example of an institution-wide platform that allows for substantial clinic-specific customization. From the outset, BRIDGE was designed by intended users who worked closely with stakeholders, through an HCD process, to develop a structured and modular solution (Figure 2) that could be scaled and customized to specific clinic use cases in a cost- and time-efficient manner (Figure 3). The resulting platform addresses clinicians’ requests to reduce data overload and more precisely tailor the data that they use during clinical encounters. The lessons learned from building an institution-wide digital medicine platform include not only the importance of using HCD but also the importance of engaging with institutional partners and leadership early to collaboratively and transparently navigate through the long and arduous process of obtaining regulatory and security approval.

Based on our experiences, we propose that the development of similar platforms at other institutions is an efficient way to accelerate the testing of digital health algorithms in clinics. To reduce the burden of this undertaking, other academic clinical centers could use all or part of the BRIDGE platform code to create their own instances, especially if these centers used Epic, even though there would still be regulatory approval and software integration steps for making BRIDGE available within their EHRs. Additional developments could simplify this further, including sharing aspects of BRIDGE through centralized application stores, such as the Epic App Orchard, as well as creating centralized security audits and certifications that allow software to be vetted thoroughly once rather than vetting software at each new institution. Such centralization could be achieved by a federal initiative, a nationwide nonprofit society, or commercial vendors. For example, commercial vendors could provide institutions with centralized platforms that provide cloud-based computational resources, data access, security, and certification while clinicians and scientists develop dashboards and algorithms that run on these centralized platforms. BRIDGE provides a way to immediately develop and test these dashboards and algorithms in preparation for this future.

The potential of precision medicine will only be realized when the utility of the algorithms developed in this field can be evaluated at the point of care with real patients. Performing this testing requires substantial infrastructure development, which is hard to justify in the evaluation phase. Modular, scalable, institution-wide platforms, such as BRIDGE, represent one approach to resolving this catch-22 problem by providing an efficient mechanism for rapidly and cost-effectively deploying and evaluating new algorithms in clinics. Such a mechanism effectively serves as a bridge for translating research innovations into clinical tools.

## Abbreviations

API: application programming interface
EHR: electronic health record
FHIR: Fast Healthcare Interoperability Resources
GUI: graphical user interface
HCD: human-centered design
HIPAA: Health Insurance Portability and Accountability Act
MVP: minimum viable product
NIH: National Institutes of Health
PACS: Picture Archiving and Communication System
REDCap: Research Electronic Data Capture
SMART: Substitutable Medical Applications and Reusable Technologies
TabCAT: Tablet-Based Cognitive Assessment Tool
UCSF: University of California, San Francisco
